# Infectious diseases during the Russian-Ukrainian war - Morbidity in the Transcarpathian region as a marker of epidemic danger on the EU border

**DOI:** 10.1016/j.puhip.2023.100397

**Published:** 2023-06-17

**Authors:** Pavlo Petakh, Aleksandr Kamyshnyi, Viktoriia Tymchyk, Richard Armitage

**Affiliations:** Department of Biochemistry and Pharmacology, Uzhhorod National University, Uzhhorod, Ukraine; Department of Microbiology, Virology, and Immunology, I. Horbachevsky Ternopil National Medical University, Ternopil, Ukraine; Department of Microbiology, Virology, and Immunology, I. Horbachevsky Ternopil National Medical University, Ternopil, Ukraine; Transcarpathian Regional Center for Disease Control and Prevention, Ukraine; University of Nottingham, Population and Lifespan Sciences, School of Medicine, United Kingdom

## Abstract

• The Transcarpathian region in Ukraine has received over 350,000 Internally Displaced Persons (IDPs) since the beginning of the invasion, making the region vulnerable to outbreaks of communicable diseases.
• The coverage of vital vaccination programs has been substantially harmed since the invasion began, with many children displaced without documentation and their vaccination status unknown.
• The conditions in which IDPs in Transcarpathia reside risk local outbreaks of infectious diseases and their transmission into EU countries, making it vital to continue supporting Ukrainian healthcare authorities on the pivotal issue of vaccination.

Dear Editor,

Nine months have passed since Russia's unprecedented invasion of Ukraine, which has triggered injuries, deaths, and displacement of people. As of 26 September 2022, an estimated 6.2 million persons – around 15% of the country's total population – are internally displaced within Ukraine [1].

The Transcarpathian region in the South-West of Ukraine - the most remote region in the country, located in the centre of Europe, bordering four EU countries, and with a population of 1.3 million people - has received over 350,000 Internally Displaced Persons (IDPs) since the beginning of the invasion (Fig. 1). A proportion of these persons have not remained in the region but moved onwards to the bordering EU countries with recognised refugee status [2].

**Fig. 1:**
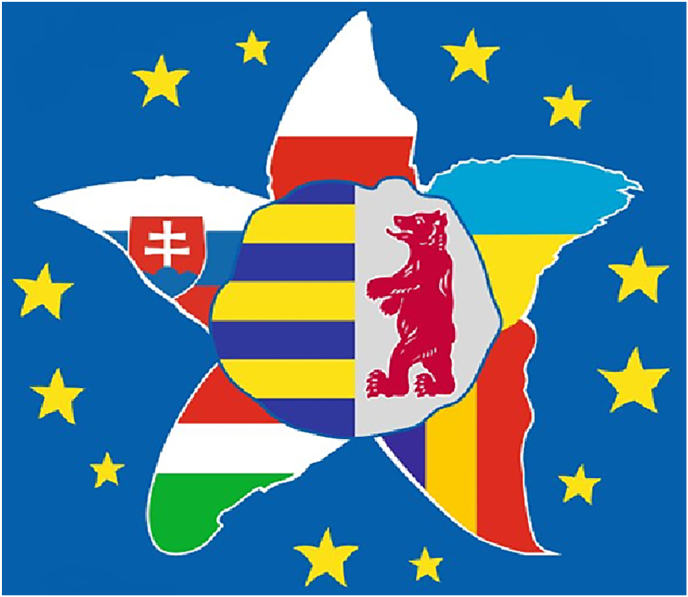
Transcarpathia's Edelweiss Flower: A Symbolic Representation of Neighboring EU States This figure depicts the symbol of Transcarpathia, which is an edelweiss flower. The coat of arms of Transcarpathia is placed inside the flower, and each petal represents a neighboring EU state that borders Transcarpathia.

The long-term health of IDPs should be of paramount concern, especially if they are housed in temporary facilities for prolonged periods. These evacuee centres are often fashioned from repurposed community buildings such as schools, nurseries, and gymnasiumss, and are routinely over-populated, under-hearted, and remotely located. The sub-population of IDPs hosted in such facilities are also those with the fewest resources. As such, they are precariously positioned and highly vulnerable to the predisposing factors to communicable diseases, which may lead to local outbreaks of infectious diseases that threaten to spread into neighboring EU countries.

Concern regarding communicable diseases in the in Transcarpathia region pre-dates the conflict's outbreak. Towards the end of 2021, a case of acute flaccid paralysis caused by cVDPV2, and several cases in asymptomatic contacts, were recorded in the region. The coverage of vital vaccination programmes has been substantially harmed since the invasion began. Millions of people have been displaced within and outside the country, making it more difficult to locate and vaccinate children. Many children have been displaced without documentation, and therefore their vaccination status is often unknown. There is also low awareness of the importance of vaccines among some hard-to-reach communities, particularly in rural areas into which IDPs have recently flooded. The overall coverage of the catch-up inactivated polio vaccine campaign in the Transcarpathian region was 35% as of 16 March 2022 [3]. Simultaneously, the incidence of measles is in danger of increasing, due to insufficient coverage in the immunization of children [4] (Ukraine experienced outbreaks of measles prior to the war, most recently in 2017–2019). Of extreme concern is the background rate of meningococcal infection in the Transcarpathia region, the incidence of which in 2016–2018 exceeded the average national indicators by several multiples [5]. The conditions in which IDPS in Transcarpathia reside risk local outbreaks of these infections and their transmission into the EU.

It is worth noting the role of EU border countries, which understand the complexity of the situation. The created cross-border cooperation program with the participation of Slovakia, Hungary, and Romania financed the construction of a microbiological laboratory on the basis of the regional infectious disease hospital, which will speed up the diagnosis of infectious diseases. Also, UNICEF financed educational work on IDP vaccination. Dr. Pavlo Kolesnyk, together with international partners, opened a clinic where vaccinations of IDPs will be carried out.

The migration and humanitarian crisis unfolding in Transcarpathia create severe risks of outbreaks of infectious diseases not only for Ukraine but also for bordering countries. Together with international partners and influential organizations, it is vital to continue to support Ukrainian healthcare authorities on the pivotal issue of vaccination.

## Conflict of interests

We declare no competing interests.
